# Annual Catalogue of the Officers and Students of the Ohio College of Dental Surgery

**Published:** 1846-09

**Authors:** 


*Annual Catalogue of the Officers and Students of the Ohio College of Dental Surgery,* session of 1846–7.

The above named institution has been in operation one year, and we are happy to learn, that the faculty, from the encouragement they have thus far received in their commendable undertaking, will enter upon the duties of their second course with renewed energy and zeal. At first it was not to be expected that they would receive an adequate compensation for their labor, but we doubt not with perseverance, and rigid requirements of those upon whom they confer the honors of their school, their efforts will ultimately be crowned with abundant success. At the conclusion of their first course of lectures, they conferred the degree of Doctor of Dental Surgery on six who had met the requirements of the institution.

Collegiate instruction is as important in the dental as in any of the other branches of medicine. It is a speciality which can only be practised successfully by those who have been thoroughly educated for it, and no one should now think of entering the profession without the requisite qualifications. And where can these be so readily and thoroughly obtained as in an institution where every thing pertaining to the science and art are taught? and not only theoretically but practically. We do not hesitate to answer, no where. Entertaining this opinion, we hope that prosperity will attend our young sister of the West, and that the faculty may never have cause to regret having engaged in so praiseworthy an enterprise.—*Balt. Ed.*

